# Infectious Dose of African Swine Fever Virus When Consumed Naturally in Liquid or Feed

**DOI:** 10.3201/eid2505.181495

**Published:** 2019-05

**Authors:** Megan C. Niederwerder, Ana M.M. Stoian, Raymond R.R. Rowland, Steve S. Dritz, Vlad Petrovan, Laura A. Constance, Jordan T. Gebhardt, Matthew Olcha, Cassandra K. Jones, Jason C. Woodworth, Ying Fang, Jia Liang, Trevor J. Hefley

**Affiliations:** Kansas State University, Manhattan, Kansas, USA

**Keywords:** African swine fever virus, ASFV, oral dose, minimum infectious dose, median infectious dose, feed, pigs, natural consumption, viruses, vector-borne infections

## Abstract

African swine fever virus (ASFV) is a contagious, rapidly spreading, transboundary animal disease and a major threat to pork production globally. Although plant-based feed has been identified as a potential route for virus introduction onto swine farms, little is known about the risks for ASFV transmission in feed. We aimed to determine the minimum and median infectious doses of the Georgia 2007 strain of ASFV through oral exposure during natural drinking and feeding behaviors. The minimum infectious dose of ASFV in liquid was 10^0^ 50% tissue culture infectious dose (TCID_50_), compared with 10^4^ TCID_50_ in feed. The median infectious dose was 10^1.0^ TCID_50_ for liquid and 10^6.8^ TCID_50_ for feed. Our findings demonstrate that ASFV Georgia 2007 can easily be transmitted orally, although higher doses are required for infection in plant-based feed. These data provide important information that can be incorporated into risk models for ASFV transmission.

African swine fever virus (ASFV) is an emerging threat to swine production in North America and Europe. During the past decade, ASFV has spread into Eastern Europe and Russia (*1*,*2*) and most recently into China (*3*,*4*) and Belgium (*5*). Disease caused by ASFV is characterized by severe disseminated hemorrhage, and case-fatality rates approach 100% (*6*). The virus is a member of the *Asfarviridae* family and is the only known vectorborne DNA virus (*7*). Challenges to disease control include the lack of available vaccines and the potential for ASFV to become endemic in feral swine and ticks (*8*). Because no effective vaccine or treatment exists, preventing ASFV introduction is the primary goal of disease-free countries. Mitigation strategies during an African swine fever (ASF) outbreak are centered around restricting pig movement and conducting large-scale culling of infected herds. It is estimated that the introduction of ASFV into the United States would cost producers >$4 billion in losses (*9*).

Historical outbreaks, including the introduction of ASFV into the Caucasus region in 2007 and subsequent spread into Russia, have been attributed to feeding contaminated pork products (*1*) or direct contact with pigs (*10*). ASFV survives in meat and blood at room temperature for several months (*11*,*12*) and is resistant to temperature and pH extremes (*13*). Molecular characterization of the more recent ASFV incursions into China (*4*) and Siberia (*14*) demonstrate similarity in viral isolates to the Georgia 2007 strain of ASFV. These outbreaks have occurred in herds separated by thousands of kilometers (*15*). For example, ASFV spread ≈2,100 km from the city Shenyang in northern China to the city Wenzhou, south of Shanghai, in ≈3 weeks (*16*). Also, an ASFV incursion has been reported recently in a large-scale, high-biosecurity farm in Romania (*17*). Contaminated water from the Danube River has been implicated in introducing ASF onto the ≈140,000-pig breeding farm (*18*). Contaminated feed as a transmission vehicle for introducing transboundary animal diseases onto high-biosecurity swine operations has been recognized as a major risk factor since the introduction of porcine epidemic diarrhea virus into the United States in 2013 (*19*–*24*). The lesson learned from porcine epidemic diarrhea virus underscores the need to quantitate the risk that feed plays in the introduction of other transboundary animal diseases. Nonetheless, data defining the risk for ASFV transmission through feed or feed ingredients are limited. 

In 2014, the introduction and spread of ASFV in Latvia was associated with the feeding of virus-contaminated fresh grass or crops to naive pigs (*25*). Furthermore, recent work has demonstrated that ASFV survives in feed ingredients, such as conventional soybean meal, organic soybean meal, soy oil cake, and choline, under conditions simulating trans-Atlantic shipment from Eastern Europe to the United States (*21*). These reports suggest that the spread of ASFV might be attributed to less-recognized transmission routes, such as feed or water.

ASFV can be transmitted experimentally through several routes, including intramuscularly, oronasally, or through direct contact (*6*). In many of the studies on oronasal transmission, however, ASFV was placed directly in the mouth or on the tonsils. The infectious dose of ASFV in plant-based feed or liquid consumed naturally is lacking; moreover, nothing has been reported regarding ASFV Georgia 2007 transmission in feed. Although field-based epidemiologic reports provide information suggesting routes of transmission, they provide little information about infectious dose. Thus, our objectives were to 1) define the relationship between infection probability and dose, 2) identify the minimum infectious dose (MID) or lowest dose required to result in ASFV infection of >1 pig, and 3) identify the median infectious dose (ID_50_) or dose required to result in ASFV infection of 50% of pigs for ASFV Georgia 2007 when consumed naturally in contaminated feed or liquid.

## Materials and Methods

### ASFV Inoculum Preparation

We used an ASFV Georgia 2007/1 isolate (*2*) for this study. Viral stocks were created from spleen tissue collected from pigs during acute infection with ASFV Georgia 2007 (*26*). We minced splenic tissue and passed it through a cell strainer in the presence of phosphate-buffered saline (PBS) supplemented with penicillin/streptomycin and fungizone. We centrifuged the suspension at 4,000 × *g* for 30 min and stored the supernatant at 4°C. We then resuspended the pellet in sterile PBS with antibiotics and antimycotics and obtained additional virus by 3 freeze-thaw cycles. The suspension was centrifuged and clarified supernatant stored at 4°C.

For virus titration, we collected porcine alveolar macrophages (PAMs) by using lung lavage of 3–5-week-old pigs. We cultured PAMs for 2 days in RPMI media supplemented with 10% fetal bovine serum and antibiotics in a 37°C 5% CO_2_ incubator. We then prepared 10-fold serial dilutions of virus in triplicate and added the dilutions to PAMs in a 96-well plate. After 3 days at 37°C, cells were fixed by using 80% acetone for 10 min. Cells were stained using a p30 monoclonal antibody (*27*) diluted 1:6,000. We incubated the plate at 37°C for 1 h and washed it 3 times with PBS. Bound antibody was detected by using a goat-anti mouse antibody (AlexaFluor 488; Thermo Fisher Scientific, https://www.thermofisher.com) diluted 1:400 and incubated for 1 h at 37°C. We observed stained cells under an inverted fluorescence microscope (Evos FL; Thermo Fisher Scientific) and calculated the log_10_ 50% tissue culture infectious dose per milliliter (TCID_50_/mL) according to the method of Reed and Muench (*28*).

 We made dilutions of the clarified ASFV Georgia 2007 splenic homogenate by using RPMI media, with doses ranging from 10^0^ TCID_50_ to 10^8^ TCID_50_ added to a final volume of 100 mL RPMI or 100 g complete feed. The feed was a typical corn soybean meal–based diet formulated to be nutritionally adequate according to the National Research Council recommendations for pigs weighing 10–25 kg (*29*). The diet did not contain any animal-based feed ingredients. For mixing virus with feed, we allowed 10 mL of virus to absorb onto 100 g of feed in a 500 mL wide-mouth high-density polyethylene round bottle (Nalgene, Thermo Fisher Scientific) for 30 s before homogenization by rolling and gently mixing the bottle by hand.

### Animals and Housing

The use of pigs and viruses in research was performed in accordance with the Federation of Animal Science Societies Guide for the Care and Use of Agricultural Animals in Research and Teaching and the US Department of Agriculture’s Animal Welfare Act and Animal Welfare Regulations. The research was approved by the Kansas State University Institutional Animal Care and Use Committee and the Institutional Biosafety Committee. 

We obtained 84 crossbred pigs (average age, 51.8 + 2.2 days) from a single high-health commercial source. Pigs were housed in 3 identical 66 m^2^ rooms at the Kansas State University Biosecurity Research Institute and maintained under Biosafety Level 3 agriculture containment conditions. Rooms were environmentally controlled, and complete exchange of air occurred 14.5 times/hour in each room. Pigs were maintained individually in 1.9 m^2^ pens, and each pen was separated by >1.5 m in the room. The stainless-steel pens were raised and contained slotted fiberglass flooring. Three sides of the pen were solid, with a fourth side consisting of bars and a gate. All efforts were made to prevent aerosol spread of virus. Negative control pigs were maintained in the room as a means to monitor the potential for cross-contamination between pens.

### Experimental Design

We adapted the experimental design and approach for determining the median infectious dose of ASFV Georgia 2007 from previous work on porcine reproductive and respiratory syndrome virus (*30*,*31*). We conducted 7 replicates for both liquid and feed, each composed of 6 pigs for liquid and 6 pigs for feed. In each replicate for feed or liquid, we administered 5 pigs a specific dose of ASFV; 1 pig served as the negative control. An adaptive study design was incorporated throughout the course of the experiment to result in the most precise estimate of the ID_50_ while maximizing the information gained from the trial (*32*,*33*). The most likely ID_50_ was based on a review of the available literature (*34*–*40*). We used this information to identify the initial infectious dose tested of 10^3^ TCID_50_ for liquid and 10^4^ TCID_50_ for feed. After completion of the first replicate, we used the continual reassessment method to update the ID_50_ estimate (*32*,*33*). The results of each replicate were used to select dosages for subsequent replicates; in general, this process resulted in liquid doses decreasing and feed doses increasing after the initial replicates were completed. All replicates and pig numbers for each dose are shown in Table 1.

**Table 1:** Replicates of pigs orally exposed to ASFV in liquid or feed based on a sequential adaptive experimental design to determine the infectious dose of ASFV when consumed naturally*

Dose ASFV, TCID_50_	Liquid media replicates, no. tested (no. positive)								Plant-based feed replicates, no. tested (no. positive)							
	1	2	3	4	5	6	7		1	2	3	4	5	6	7
10^0^	–	–	–	–	3 (3)	–	5 (0)		–	–	–	–	–	–	–
10^1^	–	–	5 (3)	5 (1)†	–	–	–		–	–	–	–	–	–	–
10^2^	–	4 (2)	–	–	2 (2)	2 (2)	–		–	–	–	–	–	–	–
10^3^	5 (5)	1 (0)	–	–	–	–	–		–	5 (0)	–	–	–	–	–
10^4^	–	–	–	–	–	3 (3)	–		5 (2)	–	–	–	–	–	–
10^5^	–	–	–	–	–	–	–		–	–	5 (2)	5 (2)†	–	–	–
10^6^	–	–	–	–	–	–	–		–	–	–	–	3 (0)	–	5 (2)
10^7^	–	–	–	–	–	–	–		–	–	–	–	2 (0)	3 (2)	–
10^8^	–	–	–	–	–	–	–		–	–	–	–	–	2 (1)	–

*Data are shown for the 5 infected pigs. In each replicate, 1 negative control pig was present. ASFV, African swine fever virus; TCID_50_, 50% tissue culture infectious dose; –, no pigs tested. †One pig in each of these replicates died before 5 days postinoculation for causes other than ASF and was eliminated from the data analysis.

For drinking, pigs consumed ASFV mixed in a 100-mL volume of RPMI media. Liquid was provided through a gravity-fed restricted-flow nipple drinker (Arato 76 Piglet Drinker; Ag Works International, http://www.agworksintl.com) attached to an adjustable galvanized wall bracket (1.3 cm × 61 cm pipe; SMB Manufacturing, https://www.smbmfg.com). If pigs became averse to drinking from a nipple, liquid medium was placed in a small stainless-steel bowl for pigs to drink. For feeding, pigs consumed ASFV mixed in a 100-g volume of complete feed provided in a 23-cm stainless-steel creep feeder (Vittetoe Inc., http://www.vittetoe.com). Infectious titers of each virus dilution were back-titrated on PAMs by endpoint titration assay (TCID_50_/mL) to confirm accurate dosing. Negative control pigs received the same volumes of sterile media or complete feed without virus.

Pigs were acclimated to the drinkers or feeders for 3–4 days before ASFV inoculation. During this acclimation period, water and feed (drinking) or feed alone (feeding) were withheld for 10–14 hours before liquid media or feed was offered. Pigs were monitored during the drinking or eating process. Once pigs had consumed the specified volume of liquid or feed, pigs were given unrestricted access to feed and water until the next withholding period. After acclimation, 5 pigs in each replicate were offered the same substrate containing a specific dose of ASFV followed by unrestricted access to feed and water.

We evaluated the pigs for clinical signs of ASF twice daily and collected blood from each pig at 0 and 5 days postinoculation (dpi). Pigs showing clinical signs before 5 dpi were humanely euthanized, and blood and tissues were collected. The remaining pigs were humanely euthanized on 5 dpi, and complete necropsies were performed. We determined infection status on the basis of real-time PCR detection of ASFV in the serum or spleen and virus isolation from the spleen. We constructed dose-response curves and calculated ID_50_, as described further in this article.

### ASFV PCR

We extracted nucleic acid from serum or splenic homogenate by using the MagMAX-96 Viral RNA Isolation Kit (Thermo Fisher Scientific). For nucleic acid isolation, we combined 50 μL of sample with 20 μL of Bead mix (containing lysis/binding solution, carrier RNA, and 100% isopropanol) on a U-bottom 96-well plate. Cells were lysed by using 130 μL lysis/binding solution and mixed for 5 minutes on a shaker. The beads were captured on a magnetic stand and washed twice using 150 μL Wash Solution 1 and 2 with a final elution volume of 50 μL.

We performed PCR amplification of p72 according to King et al. (*41*). The primer and probe mixture was commercially synthesized by using PrimeTime Mini qPCR Assay (IDT Technologies, https://www.idtdna.com): probe (5′-[6-FAM]- CCA CGG GAG ZEN GAA TAC CAA CCC AGT G-3′-[IBFQ]), sense primer (5′-CTG CTC ATG GTA TCA ATC TTA TCG A-3′), and anti-sense primer (5′-GAT ACC ACA AGA TCR GCC GT-3′). The 15 μL PCR mixture consisted of 10 μL 2X iTaq Universal Probes Supermix (Bio-Rad Laboratories, http://www.bio-rad.com), 1 μL 1X PrimeTime Mini (500 nM primers and 250 nM probe), and 4 μL nuclease-free water. We dispensed this mastermix into a Hard-Shell optical 96 well reaction plate (Bio-Rad Laboratories), added DNA samples, and briefly centrifuged the plate to remove air bubbles. We then performed real-time PCR on a CFX96 Real-Time System (Bio-Rad Laboratories) under the following conditions: 95°C for 2 min, followed by 45 cycles of 94°C for 30 s, 58°C for 1 min, and 60°C for 30 s. We performed data analysis by using CFX96 software and reported results as cycle threshold values.

### Data Analysis

We assessed infectivity by using 3 diagnostic methods (PCR of spleen, PCR of serum, and virus isolation of spleen), which resulted in 3 binary response variables (i.e., positive or negative) for each individual pig. We categorized ASFV infection as positive if >1 diagnostic test indicated evidence of infection. We analyzed all binary responses simultaneously to account for imperfect test agreement (*42*–*44*).

Without assuming a functional form for the relationship between dose and probability of infection, we used a constrained spline regression model. The constraints used were limited to the assumptions that infection probability increases as dose increases and that the relationship is continuous. We used a constrained regression spline within a Bayesian hierarchical model to estimate the infection probability at each dose for a single exposure based on the results of the 3 diagnostic methods. On the basis of the single exposure, we also modeled repeated exposures, assuming repeated exposures are independent events. Thus, we calculated the infection probability for multiple exposures as 1 – (1 – *p*)*^q^*, where *p* is the single-exposure infection probability and *q* is the number of exposures. Repeated exposures can be viewed interactively online (https://trevorhefley.shinyapps.io/asfv). We used previously described algorithms for statistical model implementation (*45*,*46*) by using the cgam package in R (*47*). We have provided a tutorial with the computational details, annotated computer code to assist readers implementing similar models, and the necessary code to reproduce results and figures related to the analysis (Appendix).

## Results

A summary of the infection results is shown in Table 2. A total of 68 pigs were included in the study. No evidence of ASFV infection was detected in the 14 negative control pigs. Therefore, adequate biosecurity was maintained throughout the study. Of the 32 pigs with evidence of ASFV infection, 16 (50%) were positive on virus isolation and PCR of spleen, 8 (25%) were positive on virus isolation of spleen alone, and 8 (25%) were positive on all 3 tests. The 34 pigs in the feeding trial consumed the 100 g of feed in a mean + SD of 14.8 + 5.5 min (minimum 7 min, maximum 30 min). For the liquid trial, the 34 pigs consumed the 100 mL of ASFV-inoculated media in a mean + SD of 21.1 + 18.2 min (minimum 3 min, maximum 63 min). A small number of pigs (3/34 [8.8%]) averse to the restricted-flow nipples consumed media from a bowl.

**Table 2:** Summary of results for pigs orally exposed to ASFV in liquid or feed to determine the infectious dose of ASFV when consumed naturally*

Dose ASFV, TCID_50_	Liquid media				Plant-based feed		
	No. tested	No. positive	% Positive		No. tested	No. positive	% Positive
10^0^	8	3	37.5		–	–	–
10^1^	9	4	44.4		–	–	–
10^2^	8	6	75		–	–	–
10^3^	6	5	83.3		5	0	0
10^4^	3	3	100		5	2	40
10^5^	–	–	–		9	4	44.4
10^6^	–	–	–		8	2	25
10^7^	–	–	–		5	2	40
10^8^	–	–	–		2	1	50

*ASFV, African swine fever virus; TCID_50_, 50% tissue culture infectious dose; –, no pigs tested.

**Figure 1:**
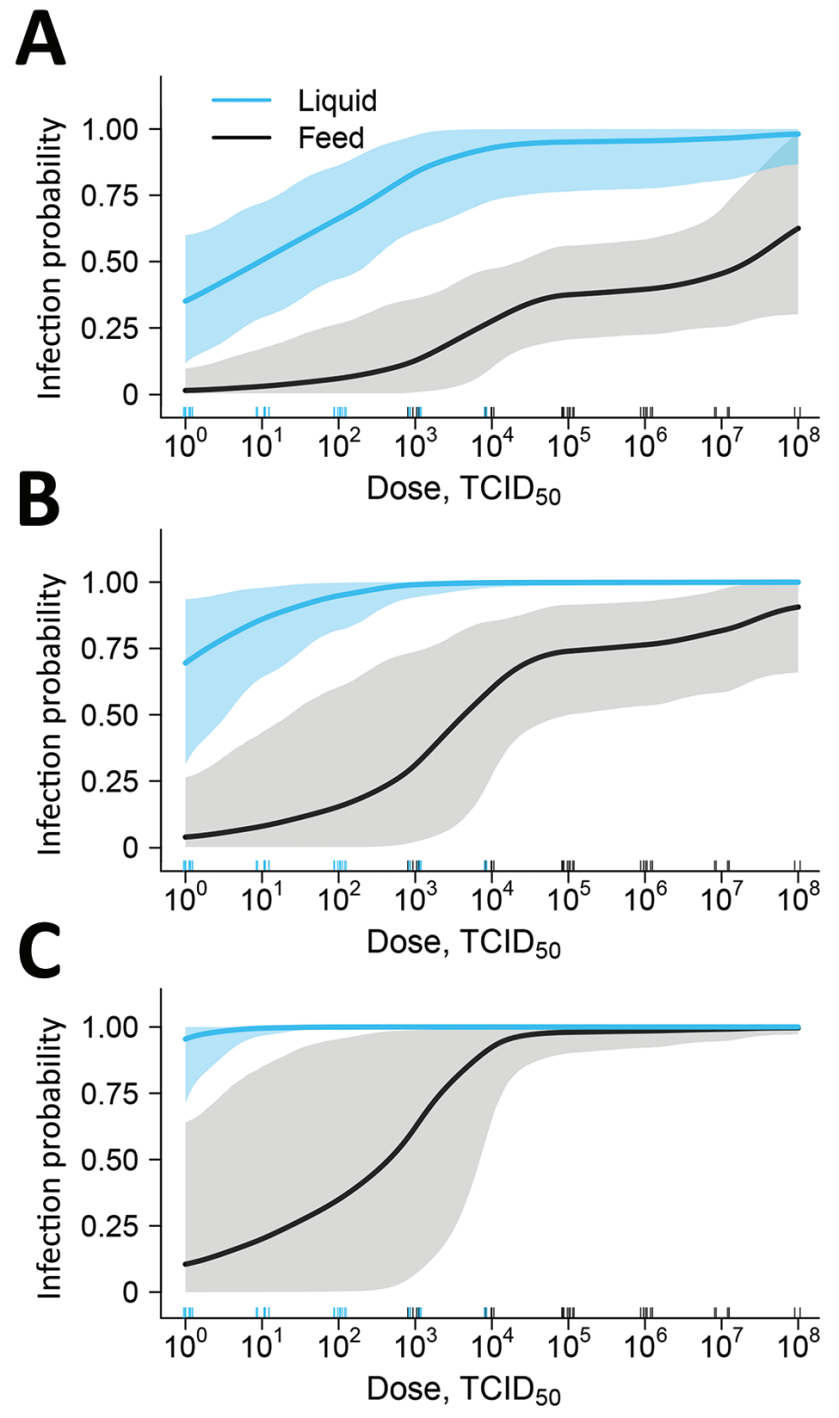
Estimated liquid (blue line) and feed (black line) infection probability at different oral doses of ASFV based on experimental data to determine the infectious dose of ASFV when consumed naturally. Data are shown for 1 exposure (A), 3 exposures (B), and 10 exposures (C). Shading indicates 95% CIs. Numbers of individual pig dosages are represented by the blue and black tick marks above the horizontal axis. Repeated exposures can be viewed interactively online (https://trevorhefley.shinyapps.io/asfv).

Overall, the probability of infection increased as the dose increased for both feed and liquid (Figure 1). Reported as the lowest dose required to result in ASFV infection of >1 pig, the MID after liquid consumption was 10^0^ TCID_50_, whereas 10^4^ TCID_50_ was the MID required to result in infection after consumption of contaminated complete feed. For a single exposure, liquid had a higher infection probability compared with feed at doses up to 10^7.5^ TCID_50_ where the 95% CIs overlap (Figure 1, panel A). At the highest dose tested in liquid (10^4^ TCID_50_)_,_ 100% of pigs were infected with ASFV; in contrast, no feed dose resulted in a 100% infection rate in this experiment.

**Figure 2:**
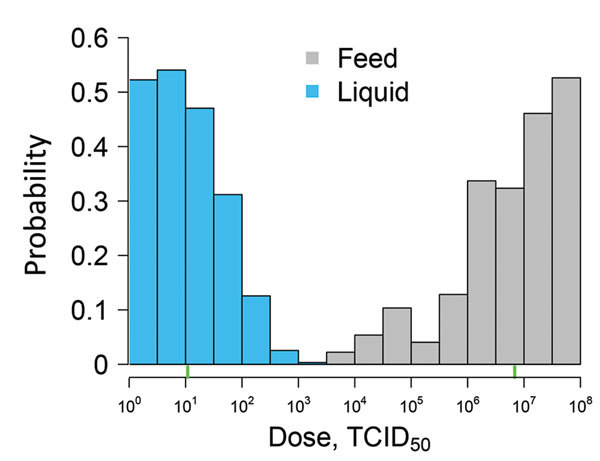
African swine fever virus (ASFV) ID_50_ distribution in a study determining the infectious dose of ASFV when consumed naturally in liquid or feed. For liquid, ID_50_ was 10^1.0^, and for feed, ID_50_ was 10^6.8^ (represented by green tick marks along baseline). ID_50_, median infectious dose (dose required to result in ASFV infection of 50% of pigs); TCID_50_, 50% tissue culture infectious dose.

When multiple exposures are considered, the infection probability increases at all dose levels for both liquid and feed (Figure 1, panels B and C). By 10 exposures with liquid, the probability of infection increases to near 1 at the lowest dose of 1 TCID_50_ ASFV. For feed with multiple exposures, we observed an increase in the width of the 95% CI at the lower dosages, indicating that with repeated exposures, the uncertainty in the infection probability increased. This result was attributable to fewer pigs being infected with lower doses and the lower infection probability for a single exposure. The distribution of plausible doses that could produce infection in 50% of pigs is shown in Figure 2. The ID_50_ was 10^1.0^ (95% CI 10^0^–10^2.3^) for liquid and 10^6.8^ (95% CI 10^4.6^–10^8+^) for feed.

## Discussion

Our study confirms the efficient transmission of ASFV by the oral route in liquid and feed lacking contaminated pork products and provides quantitative data for the Georgia 2007 strain. Early studies indicated a minimum dose of 10^5^ 50% hemadsorption doses (HAD_50_) of ASFV KWH/12 was required to cause infection when administered orally in milk (*38*). Later, Howey et al. (*35*) determined the infectious potential of 3 doses of ASFV Malawi 1983 delivered intraoropharyngeally to commercial pigs. Although a low dose of 10^2^ HAD_50_ did not induce infection (0/2), moderate (10^4^ HAD_50_) and high (10^6^ HAD_50_) doses were sufficient to cause infection in 100% of the pigs (4/4) (*35*). More recently, a study demonstrated that even lower doses of a contemporary ASFV isolate related to ASFV Georgia 2007 was capable of inducing infection. Specifically, Pietschmann et al. (*34*) showed that oronasal doses as low as 3 and 25 hemadsorption units of ASFV Armenia 2008, when delivered in 2 mL of splenic suspension, caused infection in wild boar. Increased susceptibility was demonstrated in wild boar described as weak with poor condition (*34*). 

In our study, we confirmed the high infectivity of ASFV Georgia 2007 through liquid by the oral route. Of note, the pigs in our study consumed the contaminated liquid naturally through drinking and were considered healthy and robust. Productive infection resulted in almost 40% of the pigs exposed to an ASFV liquid inoculum containing as little as 1 TCID_50_. The low infectious dose of ASFV through natural liquid consumption should be considered as a possible factor in the spread of ASF through water, consistent with the epidemiologic evidence linking the Danube River with ASF spread in Romania (*18*).

ASFV delivered through liquid by the oronasal or intraoropharyngeal route might result in infection because of virus exposure of the nasopharynx, including the tonsils, or of the gastrointestinal tract. Because of the high stability of ASFV in a wide range of pH values (from 4 to 10) (*13*), survival in the acidic gastric environment is possible but unlikely. More likely is that liquid medium provides an ideal substrate for virus contact with the tonsils, where primary virus replication occurs after natural exposure to ASFV (*38*).

Reports documenting experimental ASFV infection through contaminated feed involve consumption of tissues from infected animals. As early as 1954, it was reported that transmission of ASFV by oral feeding required a minimum dose of 10^5^ (*40*). Parker et al. failed to infect pigs with homogenized tissues from warthogs containing 10^3.7^–10^6.1^ HAD_50_ of ASFV administered in solid feed (*37*). In contrast, Colgrove et al. (*39*) successfully infected domestic pigs by adding 50 g of minced spleen and liver from an infected pig to solid feed. Each gram of tissue contained 10^7.0^–10^7.5^ HAD_50_ of ASFV isolate Hinde WH II (*39*). Our experimental studies using the contemporary isolate Georgia 2007 show that ASFV infection through the consumption of plant-based feed requires a higher dose compared with liquid. Compared with liquid media, feed might stimulate salivary proteases that degrade virus integrity. Furthermore, the feed matrix might inhibit tonsillar contact, reducing virus exposure to lymphoid and epithelial tissues before gastrointestinal entry (*36*).

Despite the higher MID in feed compared with liquid observed in this study, we hypothesize that feed might actually pose a higher risk compared with water sources in modern swine production systems. Feed delivery is a high-frequency event, and feed production is highly centralized; thus, contaminated feed can be easily distributed across a substantial number of pig farms. Pigs would also likely consume the contaminated feed in higher volumes (>100 g) and at higher frequencies (>1 exposure) than what was tested in our study. The likelihood of productive infection after consumption of ASFV-contaminated complete feed increases significantly after 3 or 10 exposures (Figure 1, panels B, C). Therefore, despite infection after consumption of ASFV in contaminated feed being a lower-probability event compared with liquid, the high frequency of exposure might make feed a more important risk factor for transmission. Adding to this risk is the fact that highly centralized feed mills use ingredients from a global distribution supply chain. For example, inventory from a midwestern US swine farm indicated feed ingredients originating from 12 countries in North America, Asia, and Europe (S.S. Dritz, unpub. data, 2018 Sep 6).

As of February 2019, ASFV had spread to a high-biosecurity farm in Romania (*17*) and had been detected in pig herds located in >25 provinces of China, including the capital Beijing (*48*), with thousands of kilometers separating affected herds. How ASFV is moving across such vast areas within the largest pork-producing country in the world is unknown; however, movement of the virus within feed or feed ingredients should be considered. The results of our study demonstrate that ASFV can be easily transmitted orally through natural consumption of both liquid and feed, supporting the potential role of feed in the emergence of this virus in new pig populations throughout the world.

Appendix Additional information on the statistical analysis to determine the probability of infection and ID_50_ of African swine fever virus when consumed naturally in liquid or feed.
