# Supplementary material for: Infectious Dose of African Swine Fever Virus When Consumed Naturally in Liquid or Feed
Source: Emerg Infect Dis. 2019 May;25(5):891–7. doi: 10.3201/eid2505.181495 (PMC6478231; doi:10.3201/eid2505.181495)
Supplement: Appendix — Additional information on the statistical analysis to determine the probability of infection and ID50 of African swine fever virus when consumed naturally in liquid or feed. [file 18-1495-Techapp-s1.pdf]

Article DOI: https://doi.org/10.3201/eid2505.181495

Infectious Dose of African Swine Fever Virus When Consumed Naturally in Liquid or Feed

Appendix

1 Introduction

This appendix provides documentation and R code associated with the analysis presented in the manuscript. The R code presented in this appendix was designed to be executed in the order presented due to dependence on prior code. We begin by loading the required R packages.

```
library(readxl)
library(stringr)
library(plotrix)
library(truncnorm)
library(cgam)
library(MASS)
library(grDevices)
library(HDInterval)
```

1.1 Data

Next we need to load the data. The data set that contains the infectivity status for each pig that was assessed using three diagnostic methods (PCR of spleen, PCR of serum, and VI of spleen) and used in this analysis is owned by Megan Niederwerder. Please contact Megan Niederwerder (mniederwerder@vet.k-state.edu) to request access to the data used in this analysis. Once the data are obtained, the R code can be used to load the data.

```
file <- "ASFV dose study.xls"
df <- read_excel(path = file, sheet = 1, range = "A1:O85")
```

Before the data are ready for analysis, we need to take several steps. The code below transforms the raw data into the format that was used for this analysis.

```
## Remove control pigs from data
df <- df[-which(df$Dose == "ctrl"), ]

# Remove pig #41 and #48, which died prior to 5 dpi
df <- df[-which(df$`Pig #` == "#48"), ]
df <- df[-which(df$`Pig #` == "#41"), ]

# Transform excel alphanumeric dose description into numerical values
df$dose <- 10^as.numeric(str_sub(gsub("[^0-9]", "", df$Dose), start = 3,
    end = 3))

# Construct a matrix that has 70 rows (one row for each pig that was
# not in the control group) and three columns (one column for each
# diagnostic method)
Y <- cbind(ifelse(df$"PCR result (spleen)" == "+", 1, 0), ifelse(df$"PCR result (serum)" ==
    "+", 1, 0), ifelse(df$"VI (spleen)" == "+", 1, 0))

# Construct a data frame that inclues the dose and type of oral dose
# (one row for each pig that was not in the control group)
X <- data.frame(dose = log10(df$dose), type = df$`Liquid/Feed`)
```

2 Data analysis

As described in the manuscript, a constrained regression spline was used within a Bayesian hierarchical model to estimate the infection probability at each dose for a single exposure based on the results of the three diagnostic methods. In what follows we explain our statistical model and algorithms used to implement our model.

2.1 Hierarchical model

Results of exposure to ASFV were assessed using three methods (PCR of spleen, PCR of serum, and VI of spleen), which resulted in three binary response variables (i.e., positive or negative) for each individual pig. Traditional analyses of these data using methods such as logistic or probit regression would require the assumption that the testing methods are 100% accurate. However, because we have multiple tests conducted on the same individual, we are able to use modern statistical methods that account

for sensitivity of the test given the time of tissue collection (Brost et al. 2018; Minuzzi-Souza et al. 2018). Although similar to traditional approaches such as logistic regression, the methods we employ appropriately account the possibility of negative test results due to the time of tissue collection, which allows for accurate estimation of the ID50 and the relationship between dose and probability of infection.

The hierarchical model that accounts for ASFV time-specific test sensitivity is also widely used in ecology and is known as the "site-occupancy model" (MacKenzie et al. 2002; Tyre et al. 2003). For our study, this model can be written as

$$[y_{ij}|p_j, z_i] = \begin{cases} \text{Bern}(p_j) & \text{if } z_i = 1 \\ 0 & \text{if } z_i = 0 \end{cases} \tag{1}$$

where the bracket notation $[a|b]$ is used to denote a conditional probability distribution (i.e., a is the random variable conditional on b), y_{ij} is equal to one for the ith pig if the jth type of ASFV diagnostic test (e.g., PCR spleen) is positive and zero if negative (note that $i = 1, 2, ..., 68$ and $j = 1, 2, 3$ for our study). The probability that the jth type of ASFV diagnostic test returns a positive result depends on the time-specific sensitivity p_j. The latent (hidden) variable z_i is the true ASFV status of the ith pig, which must be estimated from the data. A model for the true ASFV status can be written as

$$[z_i|\psi_i] = \text{Bern}(\psi_i) \tag{2}$$

where ψ_i is the probability the ith pig is truly ASFV positive. The probability that a pig is ASFV positive (i.e., ψ_i) depends on the dose and type (liquid or feed). The functional form (shape) of the relationship between dose and probability of infection is unknown, but can be written generically as

$$\psi_i = f(d_i, x_i) \tag{3}$$

where d_i is the dose administered to the ith pig via type x_i, which is either liquid or feed in this study. The function $f(\cdot)$ relates the dose to the probability of infection.

Using traditional methods, such as logistic or probit regression, the functional form, $f(\cdot)$, is assumed to be known. For example, when using logistic regression it is assumed that the "shape" of the relationship between dose and the probability of infection is the logistic function; this, however, is an assumption that is difficult to check. Instead of assuming a functional form, modern statistical methods can be used that impose relaxed assumptions on the functional form of the relationship. To do this, we embed a constrained regression spline into the so-called site-occupancy model that accounts for time-specific sensitivity (Brost et al. 2018; Minuzzi-Souza et al. 2018). To accomplish this, we use the Markov chain Monte Carlo algorithms outlined by Shaby and Fink (2012) and Dorazio and Rodriguez (2012) which enables us to embed standard software for constrained splines via the cgam package (Liao and Meyer 2018) into our hierarchical model. The constrained spline can be used to estimate the "shape" of the relationship, but only requires the assumption that the probability increases as the dose increases and that the relationship is smooth (i.e., not discontinuous).

For our analysis we take a Bayesian approach to obtain inference from our hierarchical model. Bayesian inference requires that we specify prior distributions for all unknown parameters. For p_j, we assume $p_j \sim$ unif(0,1). Implementation of the constrained spline requires that we assign a prior to an intermediate variable, f_i, explained below in section 2.2. For f_i we assume $f_i \sim \text{N}(0,\sigma_f^2)$ with σ_f^2=10.

2.2 Markov chain Monte Carlo algorithm

Markov chain Monte Carlo algorithms (MCMC) are generic algorithms that enable Monte Carlo samples to be obtained from the posterior distribution of a Bayesian hierarchical model. For our hierarchical model, we use the MCMC algorithms outlined by Shaby and Fink (2012) and Dorazio and Rodriguez (2012). Briefly, for the so-called site-occupancy model presented in Eq. 1-3, a Gibbs sampler can be constructed where all full-conditional distribution are available in closed-form (see chapter 23 in Hooten and Hefley 2019 for more details). To accomplish this, we utilize the probit link function and modify Eq. 3 so that

$$\Phi^{-1}(\psi_i) = f(d_i, x_i) \tag{4}$$

where $\Phi^{-1}(\cdot)$ is the inverse cumulative distribution function of the standard normal distribution (i.e., probit link function). This modification to our hierarchical model allows us to construct an efficient MCMC algorithm while still estimating the "shape" of the relationship between dose and the probability of that a pig is ASFV positive. The pseudocode describing our MCMC algorithm is given in Algorithm 1.

Algorithm 1. Markov chain Monte Carlo algorithm used to sample from the hierarchical Bayesian model specified by Eqs. 1–4. In this algorithm, k is the current iteration, m is the total number of iterations, and the bracket notation is used to denote a conditional distribution. The $\mathbf{Y}$ is a 68×3 matrix where the ith row contains the results of PCR of spleen, PCR of serum, and VI of spleen diagnostic methods. The $\mathbf{X}$ is a 68×2 matrix where the ith row contains the dose and method (feed or liquid) administered to the ith pig. The vector $\mathbf{p} \equiv (p_1, p_2, p_3)'$ where p_1, p_2 and p_3 is the time-specific sensitivity for PCR of spleen, PCR of serum, and VI of spleen respectively. The vector $\mathbf{z} \equiv (z_1, z_2, ..., z_{68})'$ contains the true ASFV infection status for each pig. The vector $\hat{\mathbf{f}} \equiv (\hat{f}_1, \hat{f}_2, ..., \hat{f}_{68})'$ is related to the probability that a pig is ASFV positive (ψ_i) via the inverse link function (i.e., $\psi_i = \Phi(\hat{f}_i)$). The vector $\mathbf{v}$ and $\mathbf{f}$ are intermediate variables that are used only for model implementation purposes (see Dorazio and Rodriguez 2012 and Algorithm 1 in Shaby and Fink 2012).

1: set initial values for $\mathbf{p}$ and $\hat{\mathbf{f}}$
2: **while** $k < m$ **do**
3: sample $[\mathbf{z}^k|\mathbf{p}^{k-1}, \hat{\mathbf{f}}^{k-1}, \mathbf{Y}]$
4: sample $[\mathbf{p}^k|\mathbf{z}^k, \mathbf{Y}]$
5: sample $[\mathbf{v}^k|\mathbf{z}^k, \hat{\mathbf{f}}^{k-1}]$
6: sample $[\mathbf{f}^k|\mathbf{v}^k]$
7: fit the constrained spline model to $\mathbf{f}^k$ using $\mathbf{X}$ as the predictor
8: replace $\hat{\mathbf{f}}^{k-1}$ with the predicted values of $\mathbf{f}^k$ from the contrained spline model
9: **end while**

For algorithm 1, the full-conditional distribution of z_i is

$$[z_i|\mathbf{p}, \hat{f}, \mathbf{Y}] = \begin{cases} \text{Bern}(\tilde{\psi}_i) & \text{if } \sum_{j=1}^{3} y_{ij} = 0 \\ 1 & \text{if } \sum_{j=1}^{3} y_{ij} > 0 \end{cases}$$

where

$$\tilde{\psi}_i = \frac{\psi_i(1-p_1)(1-p_2)(1-p_3)}{\psi_i(1-p_1)(1-p_2)(1-p_3) + 1 - \psi_i}.$$

The full-conditional distribution of p_j is

$$[p_j|\mathbf{z}, \mathbf{Y}] \sim \text{Beta}(a_j, b_j)$$

where $a_j = 1 + \sum_{i=1}^{68} z_i y_{ij}$ and $b_j = 1 + \sum_{i=1}^{68} z_i(1 - y_{ij})$. The full-conditional distribution of v_i is

$$[v_i|z_i, \hat{f}_i] \sim \begin{cases} \text{TN}(\hat{f}_i, 1)_0^{\infty} & \text{if } z_i = 1 \\ \text{TN}(\hat{f}_i, 1)_{-\infty}^{0} & \text{if } z_i = 0 \end{cases}$$

where $\text{TN}(\mu, \sigma^2)_a^b$ refers to a truncated normal distribution with parameters μ and σ^2 that is truncated below at a and above at b. The full-conditional distribution for f_i is

$$[f_i|v_i] \sim \text{N}(qv_i, q)$$

where $q = \frac{\sigma_f^2}{\sigma_f^2+1}$ and σ_f^2 is the prior variance for f_i (see Shaby and Fink 2012). Below is the R code implementing Algorithm 1.

```
MCMC <- function(Y,X,model.formula,sigma2.f,p.start,iter,seed,newdata){

  # Preliminary steps
  ptm <- proc.time()
  set.seed(seed)
  samples.pred <- matrix(,iter + 1,dim(newdata)[1])
  samples.p <- matrix(,iter + 1,dim(Y)[2])
  n <- dim(Y)[1]
  J <- dim(Y)[2]

  # Set initial values (line 1 of Algorithm 1)
  f.hat <- 0
  samples.p[1,] <- p.start
  p <- samples.p[1,]

  # Obtain MCMC samples (line 2 of Algorithm 1)
for (k in 1:iter) {

  # Sample z (line 3 of Algorithm 1)
  psi <- pnorm(f.hat)
  z <- ifelse(rowSums(Y)>0,1,rbinom(n,1,(psi*prod(1-p))/(psi*prod(1-p)+1-psi)))

  # Sample p (line 4 of Algorithm 1)
  p <- rbeta(J,1+colSums(Y[which(z==1),]),1+colSums((1-Y)[which(z==1),]))

  # Sample v (line 5 of Algorithm 1)
  v <- rtruncnorm(n, a = ifelse(z == 0, -Inf, 0), b = ifelse(z == 1, Inf, 0),mean = f.hat, sd = 1)

  # Sample f (line 6 of Algorithm 1)
```

```
  X$f <- rnorm(n,(sigma2.f/(sigma2.f+1))*v,(sigma2.f/(sigma2.f+1))^0.5)

  # fit constrained spline model to f using d and type (liquid or feed) as the predictor (line 7 of Algorithm 1)
  f.star <-  cgam(model.formula,data=X)

  #replace f.hat with the predicted values of f from the contrained spline model (line 8 of Algorithm 1)
  f.hat <- predict(f.star)$fit

  # Save posterior samples and print iteration number
  samples.pred[k + 1,] <- pnorm(predict(f.star,newdata)$fit)
  samples.p[k + 1,] <- p
  if(k%%1000==0)print(k)
}
print((proc.time() - ptm)[1])
list(f = samples.pred,p = samples.p)
}
```

2.3 Sampling from the posterior distribution

Using a single chain, we draw 10,000 samples from the posterior distribution using the function `MCMC(...)` that was created in section 2.2. For reference, it takes about 7 minutes to obtain 20,000 samples using the MCMC algorithm on a desktop computer equipped with a ten-core 4.5 GHz processor, 128 GB of RAM, and optimized basic linear algebra subprograms.

```
# new data frame to obtain samples of expected value of posterior preditive
# distribution
newdata <- data.frame(dose = rep(seq(0, 8, by = 0.05), 2))
newdata$type <- rep(unique(X$type), each = dim(newdata)[1]/2)

samples <- MCMC(Y = Y, X = X, model.formula = as.formula(f ~ type + s.incr(dose,
    knots = 0:8)), sigma2.f = 10, p.start = 0.5, iter = 20000, seed = 1539, newdata = newdata)

[1] 1000
[1] 2000
[1] 3000
[1] 4000
[1] 5000
[1] 6000
[1] 7000
[1] 8000
[1] 9000
[1] 10000
[1] 11000
[1] 12000
[1] 13000
[1] 14000
[1] 15000
[1] 16000
[1] 17000
[1] 18000
[1] 19000
[1] 20000
user.self
  421.396
```

After obtaining the samples it is important to check the MCMC algorithm for convergence and to determine the appropriate burn-in interval (see Hooten and Hefley 2019 for more details). Trace plots, which were the diagnostics we used to check convergence the MCMC algorithm, can be obtained with the code below (plots not shown).

```
# MCMC checks
plot(samples$p[,1],typ="l",xlab="k",ylab=expression(p[1]))
plot(samples$p[,2],typ="l",xlab="k",ylab=expression(p[2]))
plot(samples$p[,3],typ="l",xlab="k",ylab=expression(p[3]))

dose <- 1
```

```
type <- "feed"
plot(samples$f[,which(newdata$dose == dose & newdata$type == type)],typ="l",xlab="k",ylab="E(y_new|y)")
```

2.4 Figure 1

Below is the R code to create figure 1.

```
# pdf(file='Fig_1.pdf',width = 5, height=7)
par(mfcol = c(3, 1))
par(mar = c(0, 2, 0, 0), oma = c(4.1, 3.7, 2.1, 2.1))

burn.in <- 1000
n.expos <- 1
f.bar <- colMeans(1 - (1 - samples$f[-c(1:burn.in), which(newdata$type == "feed")])^n.expos)
plot(newdata$dose[which(newdata$type == "feed")], f.bar, typ = "l", lwd = 3, ylim = c(-0.05,
    1.15), xlab = "Dose", ylab = "Probability of infection", xaxt = "n", yaxt = "n",
    cex.lab = 1.4)
f.CI <- apply(1 - (1 - samples$f[-c(1:burn.in), which(newdata$type == "feed")])^n.expos,
    2, FUN = quantile, prob = c(0.025, 0.975))
polygon(c(newdata$dose[which(newdata$type == "feed")], rev(newdata$dose[which(newdata$type ==
    "feed")])), c(f.CI[1, ], rev(f.CI[2, ])), col = rgb(0.5, 0.5, 0.5, 0.3), border = NA)
f.bar <- colMeans(1 - (1 - samples$f[-c(1:burn.in), which(newdata$type == "liquid")])^n.expos)
points(newdata$dose[which(newdata$type == "liquid")], f.bar, typ = "l", lwd = 3,
    col = "deepskyblue", ylim = c(0, 1), xlab = "Dose", ylab = "Probability of infection")
f.CI <- apply(1 - (1 - samples$f[-c(1:burn.in), which(newdata$type == "liquid")])^n.expos,
    2, FUN = quantile, prob = c(0.025, 0.975))
polygon(c(newdata$dose[which(newdata$type == "liquid")], rev(newdata$dose[which(newdata$type ==
    "liquid")])), c(f.CI[1, ], rev(f.CI[2, ])), col = adjustcolor("deepskyblue",
    alpha.f = 0.3), border = NA)
axis(2, seq(0, 1, by = 0.25), las = 1, cex.axis = 1.7)
legend(x = 0, y = 1.25, cex = 1.7, legend = c("Liquid", "Feed"), bty = "n", lty = 1,
    lwd = 2, col = c("deepskyblue", "black"))
mtext("a", cex = 1.2, side = 2, line = -1, at = 1.14, las = 2)

n.expos <- 3
f.bar <- colMeans(1 - (1 - samples$f[-c(1:burn.in), which(newdata$type == "feed")])^n.expos)
plot(newdata$dose[which(newdata$type == "feed")], f.bar, typ = "l", lwd = 3, ylim = c(-0.05,
    1.15), xlab = "Dose", ylab = "Probability of infection", xaxt = "n", yaxt = "n",
    cex.lab = 1.4)
f.CI <- apply(1 - (1 - samples$f[-c(1:burn.in), which(newdata$type == "feed")])^n.expos,
    2, FUN = quantile, prob = c(0.025, 0.975))
polygon(c(newdata$dose[which(newdata$type == "feed")], rev(newdata$dose[which(newdata$type ==
    "feed")])), c(f.CI[1, ], rev(f.CI[2, ])), col = rgb(0.5, 0.5, 0.5, 0.3), border = NA)
f.bar <- colMeans(1 - (1 - samples$f[-c(1:burn.in), which(newdata$type == "liquid")])^n.expos)
points(newdata$dose[which(newdata$type == "liquid")], f.bar, typ = "l", lwd = 3,
    col = "deepskyblue", ylim = c(0, 1), xlab = "Dose", ylab = "Probability of infection")
f.CI <- apply(1 - (1 - samples$f[-c(1:burn.in), which(newdata$type == "liquid")])^n.expos,
    2, FUN = quantile, prob = c(0.025, 0.975))
polygon(c(newdata$dose[which(newdata$type == "liquid")], rev(newdata$dose[which(newdata$type ==
    "liquid")])), c(f.CI[1, ], rev(f.CI[2, ])), col = adjustcolor("deepskyblue",
    alpha.f = 0.3), border = NA)
axis(2, seq(0, 1, by = 0.25), las = 1, cex.axis = 1.7)
mtext("b", cex = 1.2, side = 2, line = -1, at = 1.14, las = 2)

n.expos <- 10
f.bar <- colMeans(1 - (1 - samples$f[-c(1:burn.in), which(newdata$type == "feed")])^n.expos)
plot(newdata$dose[which(newdata$type == "feed")], f.bar, typ = "l", lwd = 3, ylim = c(-0.05,
    1.15), xlab = "Dose", ylab = "Probability of infection", xaxt = "n", yaxt = "n",
    cex.lab = 1.4)
f.CI <- apply(1 - (1 - samples$f[-c(1:burn.in), which(newdata$type == "feed")])^n.expos,
    2, FUN = quantile, prob = c(0.025, 0.975))
polygon(c(newdata$dose[which(newdata$type == "feed")], rev(newdata$dose[which(newdata$type ==
    "feed")])), c(f.CI[1, ], rev(f.CI[2, ])), col = rgb(0.5, 0.5, 0.5, 0.3), border = NA)
f.bar <- colMeans(1 - (1 - samples$f[-c(1:burn.in), which(newdata$type == "liquid")])^n.expos)
```

```
points(newdata$dose[which(newdata$type == "liquid")], f.bar, typ = "l", lwd = 3,
    col = "deepskyblue", ylim = c(0, 1), xlab = "Dose", ylab = "Probability of infection")
f.CI <- apply(1 - (1 - samples$f[-c(1:burn.in), which(newdata$type == "liquid")])^n.expos,
    2, FUN = quantile, prob = c(0.025, 0.975))
polygon(c(newdata$dose[which(newdata$type == "liquid")], rev(newdata$dose[which(newdata$type ==
    "liquid")])), c(f.CI[1, ], rev(f.CI[2, ])), col = adjustcolor("deepskyblue",
    alpha.f = 0.3), border = NA)
axis(2, seq(0, 1, by = 0.25), las = 1, cex.axis = 1.7)
mtext("c", cex = 1.2, side = 2, line = -1, at = 1.14, las = 2)

set.seed(3405)
rug(jitter(log10(df$dose[which(df$`Liquid/Feed` == "feed")]), 1/2))
rug(jitter(log10(df$dose[which(df$`Liquid/Feed` == "liquid")]), 1/2, ), col = "deepskyblue")
axis(1, c(0:8), labels = c(expression(10^0), expression(10^1), expression(10^2),
    expression(10^3), expression(10^4), expression(10^5), expression(10^6), expression(10^7),
    expression(10^8)), cex.axis = 1.7)
mtext(expression("Dose (TCI" * D[50] * ")"), side = 1, outer = TRUE, cex = 1.2, line = 3.2)
mtext("Infection Probability", side = 2, outer = TRUE, cex = 1.2, line = 2)
# dev.off()
```

2.5 Figure 2 & ID_{50}

Below is the R code to create figure 2, which shows the posterior distribution of the median infections dose (ID_{50}).

```
# pdf(file='Fig_2.pdf',width = 7, height=5)
burn.in <- 1000
par(mar = c(5, 5, 2, 2))
temp <- apply(samples$f[-c(1:burn.in), which(newdata$type == "feed")], 1, FUN = function(x) {
    which(abs(x - 0.5) == min(abs(x - 0.5)))
})
hist(newdata$dose[unlist(lapply(temp, median))], cex.lab = 1.4, col = "grey", xlim = c(-0.5,
    8.5), freq = FALSE, xlab = expression("Dose (TCI" * D[50] * ")"), ylab = expression("Probability (I" *
    D[50] * ")"), main = "", breaks = seq(0, 8, by = 0.5), ylim = c(0, 0.6), xaxt = "n",
    las = 1, cex.axis = 1.7, cex.lab = 1.7)
rug(mean(newdata$dose[unlist(lapply(temp, median))]), col = "green", lwd = 3)

temp <- apply(samples$f[-c(1:burn.in), which(newdata$type == "liquid")], 1, FUN = function(x) {
    which(abs(x - 0.5) == min(abs(x - 0.5)))
})
hist(newdata$dose[unlist(lapply(temp, median))], col = "deepskyblue", xlim = c(-0.5,
    8.5), freq = FALSE, xlab = "Dose", ylab = expression("Probability (I" * D[50] *
    ")"), main = "", add = TRUE, breaks = seq(0, 8, by = 0.5))
axis(1, c(0:8), labels = c(expression(10^0), expression(10^1), expression(10^2),
    expression(10^3), expression(10^4), expression(10^5), expression(10^6), expression(10^7),
    expression(10^8)))
legend("top", cex = 1.7, legend = c("Feed", "Liquid"), bty = "n", pch = 15, col = c("grey",
    "deepskyblue"))
rug(mean(newdata$dose[unlist(lapply(temp, median))]), col = "green", lwd = 3)
# dev.off()
```

In addition to displaying the posterior distribution of the ID_{50} in figure 2, we report the mean and 95% credible intervals of the posterior distributions. These summaries of the posterior distribution can be obtained with the code below.

```
# Summary of posterior distribution of the ID50 for feed
temp <- apply(samples$f[-c(1:burn.in), which(newdata$type == "feed")], 1, FUN = function(x) {
    which(abs(x - 0.5) == min(abs(x - 0.5)))
})
mean(newdata$dose[unlist(lapply(temp, median))])

[1] 6.83498

hdi(newdata$dose[unlist(lapply(temp, median))], credMass = 0.95)

lower upper
```

```
  4.6   8.0
attr(,"credMass")
[1] 0.95

# Summary of posterior distribution of the ID50 for liquid
temp <- apply(samples$f[-c(1:burn.in), which(newdata$type == "liquid")], 1, FUN = function(x) {
    which(abs(x - 0.5) == min(abs(x - 0.5)))
})
mean(newdata$dose[unlist(lapply(temp, median))])

[1] 1.037322

hdi(newdata$dose[unlist(lapply(temp, median))], credMass = 0.95)

lower upper
 0.00  2.25
attr(,"credMass")
[1] 0.95
```

Finally, we also reported the dose (for a single exposure) where liquid and feed may have the same probability of infection. This dose can be identified visually from figure 2a as the point where the lower limit of the 95% credible interval for liquid touches the upper limit of the 95% credible interval for feed. The code below calculates this point directly.

```
# Dose in Fig. 1a were CI for liquid and feed meet
temp <- which(apply(1 - (1 - samples$f[-c(1:burn.in), which(newdata$type == "liquid")])^n.expos,
    2, FUN = quantile, prob = c(0.025)) < apply(1 - (1 - samples$f[-c(1:burn.in),
    which(newdata$type == "feed")])^n.expos, 2, FUN = quantile, prob = c(0.975)))
min(newdata$dose[which(newdata$type == "liquid")][temp])

[1] 7.5
```

References

Brost BM, Mosher BA, Davenport KA. A model-based solution for observational errors in laboratory studies. Mol Ecol Resour. 2018;18:580–9. PubMed http://dx.doi.org/10.1111/1755-0998.12765

Dorazio RM, Rodriguez DT. A Gibbs sampler for Bayesian analysis of site-occupancy data. Methods Ecol Evol. 2012;3:1093–8. http://dx.doi.org/10.1111/j.2041-210X.2012.00237.x

Hooten MB, Hefley TJ. Bringing Bayesian models to life. Boca Raton (FL): Chapman and Hall/CRC; 2019.

Liao X, Meyer MC. cgam: an R package for the constrained generalized additive model [cited 2018 Dec 21]. https://arxiv.org/abs/1812.07696

MacKenzie DI, Nichols JD, Lachman GB, Droege S, Andrew Royle J, Langtimm CA. Estimating site occupancy rates when detection probabilities are less than one. Ecology. 2002;83:2248–55. http://dx.doi.org/10.1890/0012-9658(2002)083[2248:ESORWD]2.0.CO;2

Minuzzi-Souza TTC, Nitz N, Cuba CAC, Hagström L, Hecht MM, Santana C, et al. Surveillance of vector-borne pathogens under imperfect detection: lessons from Chagas disease risk (mis)measurement. Sci Rep. 2018;8:151. PubMed http://dx.doi.org/10.1038/s41598-017-18532-2

Shaby BA, Fink D. Embedding black-box regression techniques into hierarchical Bayesian models. J Stat Comput Simul. 2012;82:1753–66. http://dx.doi.org/10.1080/00949655.2011.594052

Tyre AJ, Tenhumberg B, Field SA, Niejalke D, Parris K, Possingham HP. Improving precision and reducing bias in biological surveys: estimating false-negative error rates. Ecol Appl. 2003;13:1790–801. http://dx.doi.org/10.1890/02-5078
